# Untargeted serum metabonomics study of psoriasis vulgaris based on ultra-performance liquid chromatography coupled to mass spectrometry

**DOI:** 10.18632/oncotarget.21562

**Published:** 2017-10-06

**Authors:** Li Li, Lu Chuan-Jian, Han Ling, Deng Jing-Wen, He Ze-Hui, Yan Yu-Hong, Zhang Zhong-Zhao

**Affiliations:** ^1^ Molecular Biology and Systems Biology Team of Chinese Medicine, Guangdong Provincial Hospital of Chinese Medicine, Guangdong Provincial Academy of Chinese Medical Sciences, Guangzhou 510120, China; ^2^ Department of Dermatology, Guangdong Provincial Hospital of Chinese Medicine, Guangdong Provincial Academy of Chinese Medical Sciences, Guangzhou 510120, China; ^3^ Large Data Research Team of Chinese Medicine, Guangdong Provincial Hospital of Chinese Medicine, Guangdong Provincial Academy of Chinese Medical Sciences, Guangzhou 510120, China

**Keywords:** psoriasis, metabonomics, LC-MS, biomarker, high-throughput

## Abstract

Psoriasis is a common, chronic, systemic inflammatory skin disease, the etiology and pathogenesis is unclear. An untargeted high-throughput metabonomics method based on liquid chromatography coupled to mass spectrometry was applied to study the serum metabolic changes in psoriasis vulgaris patients, and to discover serum potential biomarkers for identification, diagnosis and exploring pathogenesis of psoriasis. The serum metabolic profiles from 150 subjects (75 psoriasis patients and 75 healthy controls) were acquired, the raw spectrometric data were processed by multivariate statistical analysis, and 44 potential biomarkers were screened out and identified. The potential biomarkers were mainly involved in glycerophospholipid metabolism, sphingolipid metabolism, arachidonic acid metabolism, bile acid biosynthesis, indicated the pathogenesis of psoriasis may be related to the disturbed metabolic pathways.

## INTRODUCTION

Psoriasis is a common, chronic, systemic inflammatory skin disease, the incidence and prevalence of psoriasis is significantly varied in different population, the prevalence in adults range from 0.91% (United States) to 8.5% (Norway), affecting approximately 2% of the population [1–3]. Patients with psoriasis are embarrassed about the appearance of their skin, involved in psychiatric comorbidity and suicide risk [4]. Psoriasis is considered a multisystem disease, the recent research results show it is associated with several comorbidities, such as inflammatory bowel disease, psychiatric disorders, metabolic syndrome and cardiovascular disease, as well as some new comorbidities in osteoporosis, obstructive sleep apnea and chronic obstructive pulmonary disease were reported in current literature [5]. Multiple epidemiologic studies and clinical researches have revealed that there was high prevalence of metabolic syndrome in patients with psoriasis compared with the general population or other skin diseases, and patients with more severe psoriasis have greater odds of metabolic syndrome than those with milder psoriasis. Psoriasis is associated with several metabolic diseases, including obesity, hypertension, diabetes mellitus, dyslipidemia, ischemic heart disease [6–11].

Psoriasis is a genetically determined by multiple genes, multi-environment factors stimulated, immune abnormalities induced, chronic inflammatory skin disease [12–14]. However, the etiology and pathogenesis of psoriasis is unclear, mainly associated with the genetic, immune, infection, metabolic disorders, endocrine, nervous and mental factor [15]. Metabolic abnormalities in patients with psoriasis had received extensive attention, involving proteins, lipids, carbohydrates, vitamins and enzymes and other metabolic disorders [16–23].

In recent years, research on etiology and pathogenesis of psoriasis mostly focus on immunological mechanisms and signaling pathways, the molecular mechanisms of psoriasis have been elucidated, the evidences of the relationship between psoriasis and metabolic syndrome come from clinical analysis or epidemiological analysis [24–26], however, only several studies dedicate to explore the global metabolic profiling of psoriasis patients [27–29].

Metabonomics is a modern high-throughput technique, it is an important component of systems biology, provides quantitative measures of global changes in the metabolic profiles of individuals in response to pathophysiological stimuli or genetic modification [30–32]. Profiling of metabolic products (metabolic phenotyping or metabotyping) has provided new insights into metabolic syndrome [33]. The metabonomics method has been applied to the diseases diagnosis [34], drug efficacy and toxicity evaluation [35–37], and disease pathogenesis exploration [38].

In this research, we used the ultra-performance liquid chromatography coupled with quadruple time of flight mass spectrometry (UPLC/Q-TOF MS) - based metabonomics strategy to investigate and compare the metabolic changes in the psoriasis vulgaris patients and healthy controls, as well as to discover the psoriasis biomarkers by identifying significantly changed metabolites.

## RESULTS

### Metabolic profiling of the psoriasis vulgaris patient and healthy control were established by UPLC/Q-TOF MS

**Figure 1:**
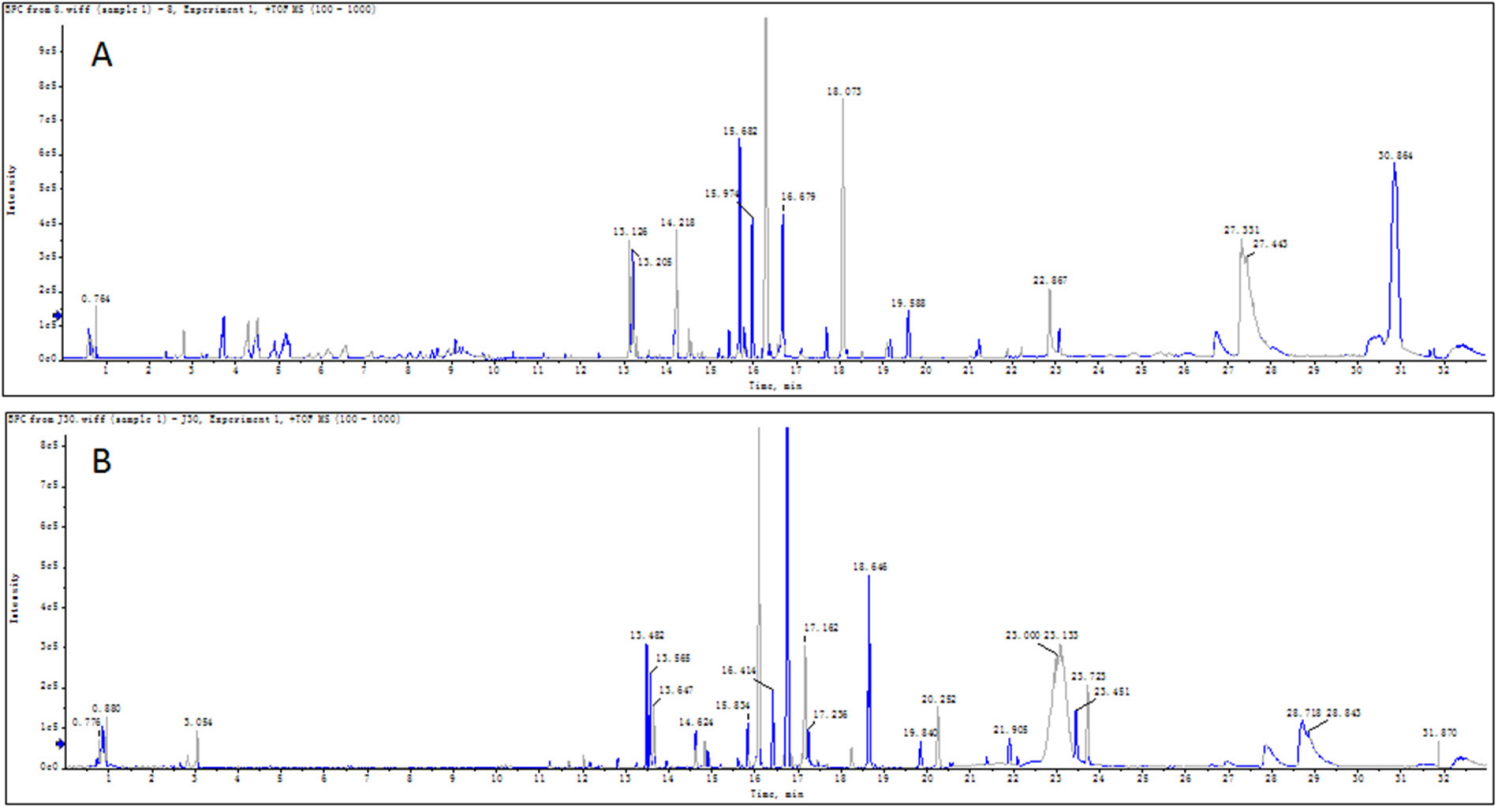
Typical base peak chromatograms (BPC) of serum sample The typical base peak chromatograms based on UPLC/Q-TOF MS in positive ion mode of psoriasis patient **(A)** and healthy control **(B)** was shown respectively.

The metabonomics study based on UPLC/Q-TOF MS was applied to investigate the serum metabolic profiling of the psoriasis patients and healthy controls. The typical serum base peak chromatograms in positive ion mode were shown in Figure 1.

### Methodology investigation

The quality control (QC) sample obtained by mixing 20μL serum of each group of sample, processed as sample preparation. One QC sample was injected into LC-MS after every 10 serum samples running to ensure the repeatability and stability in the metabonomics raw data acquisition. The relative standard deviations (RSD) of the retention times of the main peaks was less than 1% and RSD of the main peak area corresponding value was less than 10% in the QC samples. The results showed that the metabonomics method had a good repeatability, the instrument stability was excellent, and the acquired data were reliable as well.

### Multivariate statistical analysis

**Figure 2:**
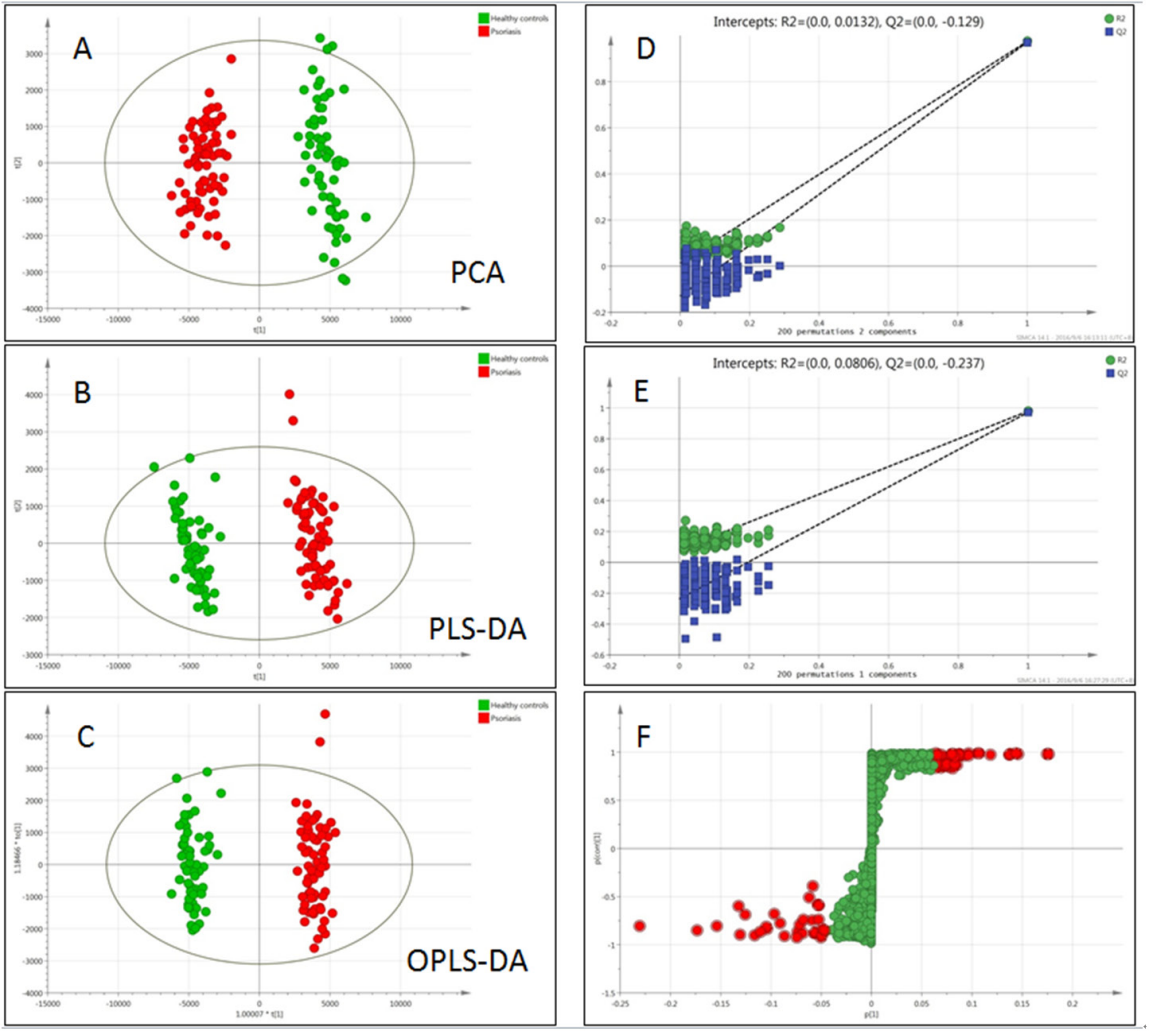
Multivariate data analysis and permutation test of psoriasis and healthy controls **(A)** PCA scores plots, **(B)** PLS-DA, **(C)** OPLS-DA(● PV patients, ● HC), **(D)** Permutation test of PLS-DA, **(E)** Permutation test of OPLS-DA, **(F)** S-plots: The variables of the maximus VIP values were marked red. The normalized data were imported to Simca-P for multivariate statistical analysis. These models showed excellent predictive ability, and validated to be no over fitting by permutation test with 200 iterations. The red marked plots in the S-plot of PV and HC were significant different variables, and considered as the biomarker candidates.

After the raw data were acquired and processed, 5220 peaks were detected. The normalized data were imported to Simca-P for multivariate statistical analysis. Both unsupervised (PCA) and supervised (PLS-DA, OPLS-DA) multivariate data analysis methods were applied to build the prediction models and classify the different groups. As an unbiased statistical approach, PCA was first carried out to distinguish the metabolic difference between PV and HC. Figure 2A showed the PCA score plots, there were distinct trends of separation between PV group and HC group indicated the inherent metabolic changes of PV individuals compared with the HC individuals. The PCA model showed the 17 principle components could explained 97.4% of the variation in the metabolic profiling [R2X(cum)=0.974] with a predictability of 91.2% [Q2(cum)=0.912], it was an excellent model. To further reveal the differences between PV and HC to discover significant variables, PLS-DA as a supervised method was applied. Figure 2B showed a clear separation in the PLS-DA model between the two groups. The parameters demonstrated it was a satisfactory modeling, the [R2X(cum)=0.774, R2Y(cum)=0.975, Q2(cum)=0.971], this model predictive ability was excellent, and validated to be no over fitting by permutation test with 200 iterations (Figure 2D). The OPLS-DA model also validated to be no over fitting by permutation test (Figure 2E).

**Figure 3:**
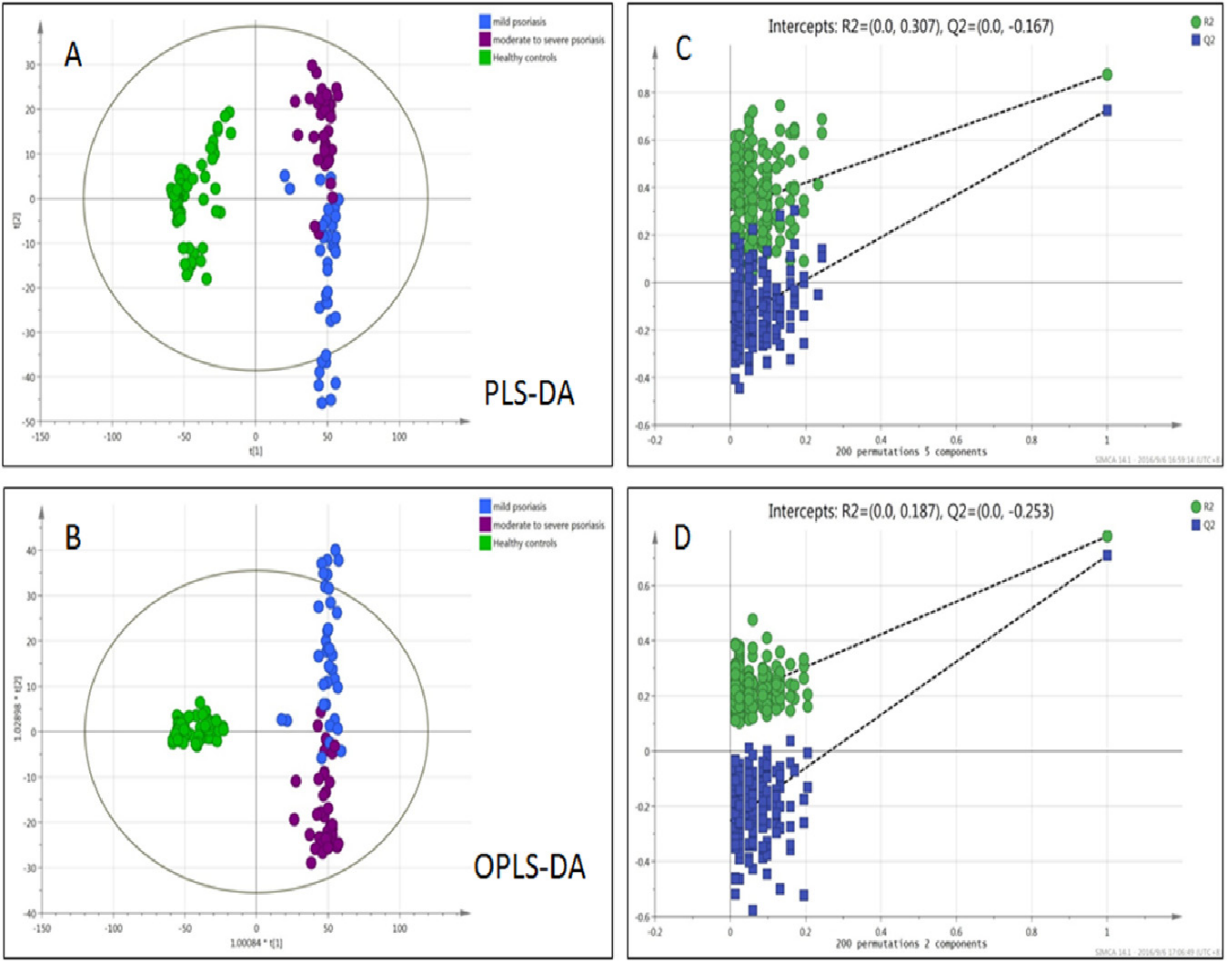
Multivariate data analysis and permutation test of psoriasis in varying degrees of severity **(A)** PLS-DA scores plots, **(B)** OPLS-DA, **(C)** Permutation test of PLS-DA, **(D)** Permutation test of OPLS-DA (● mild PV patients, ● moderate to severe PV patients, ● HC). The severity of psoriasis is defined by PASI score, PASI ≦10 being considered mild, PASI >10 being considered moderate to severe, compared with the healthy control, statistical pattern recognition results were shown.

The severity of psoriasis is defined by PASI score, PASI ≦10 being considered mild, PASI >10 being considered moderate to severe, compared with the healthy control, statistical pattern recognition results were shown in Figure 3. Significant differences in metabolic status were observed among mild psoriasis group, moderate to severe psoriasis group, and healthy controls group, the three groups were clearly clustering, indicated that the severity of psoriasis was associated with the metabolism changes in serum. Thus it might be the key point of psoriasis occurrence and development process, we could discover the potential biomarkers to diagnose and cure psoriasis with more efficiency.

### Potential biomarker screening and identification

According to the PLS-DA pattern recognition result, the Variable Importance in the Projection (VIP) values were sorted from small to large, the greater the value of grouping contribution is larger, combined with t-test, the significant difference (P < 0.05) metabolites between the psoriasis patients group and healthy controls group can be screened out. In order to identify potential biomarkers among thousands of variables, an S-plot (Figure 2F) model was utilized, and the OPLS-DA model score plots [R2(cum)=0.814, R2Y(cum)=0.979, Q2(cum)=0.972] were showed in Figure 2C. The S-plot of PV and HC was shown in Figure 2F, the marked plots were significant different variables, and considered as the biomarker candidates.

The TOF-MS can provide precise molecular weight of the screened metabolites, then search the potential biomarker in the human metabolome database (HMDB), combined with isotopic molecular weight, mass spectrometry ionization fragmentation patterns and biological significance, the potential biomarkers can be identified.

**Figure 4:**
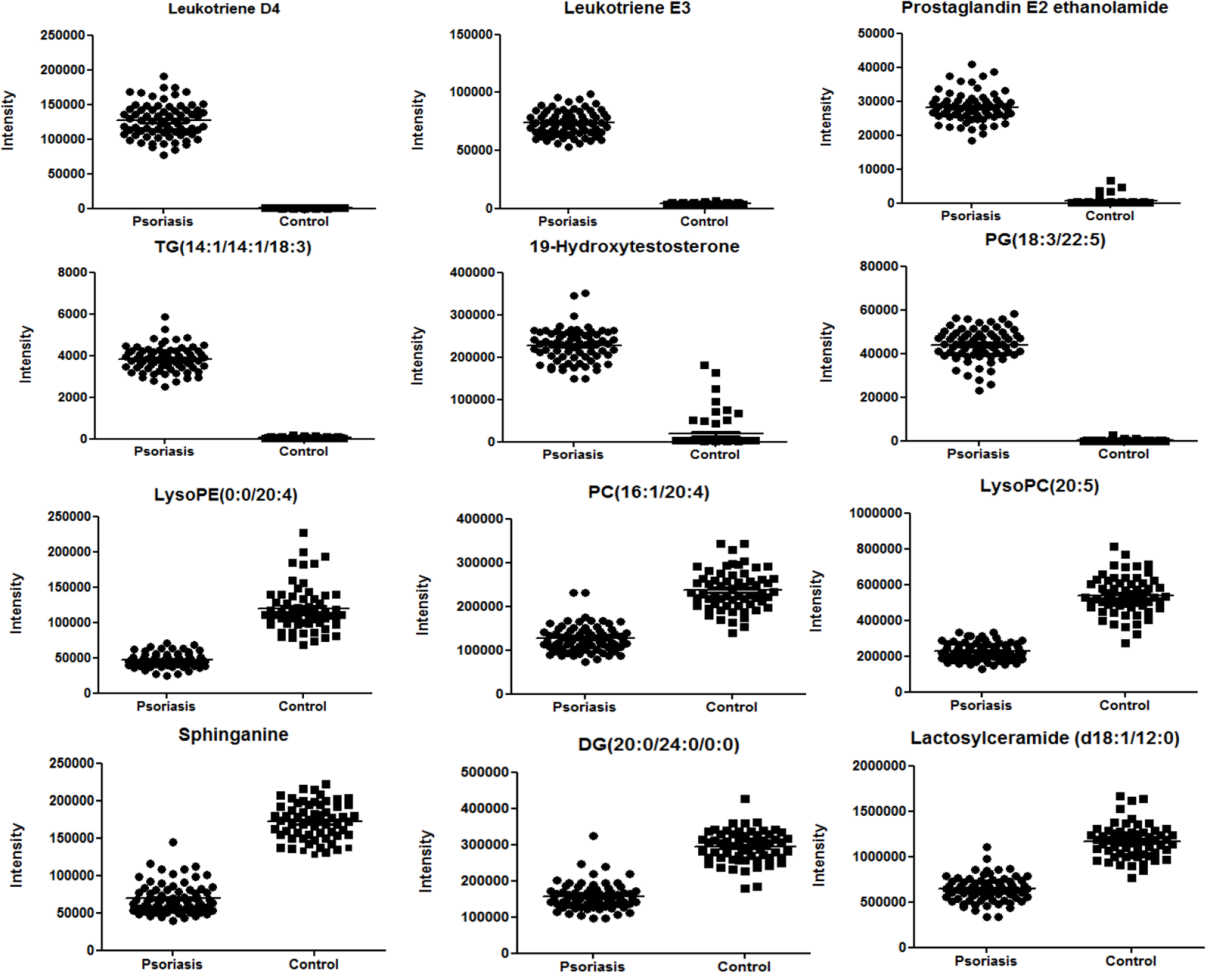
Typical metabolites with significant alterations in psoriasis vs. control The peaks intensities of potential biomarkers in serum of the two groups were shown.

**Figure 5:**
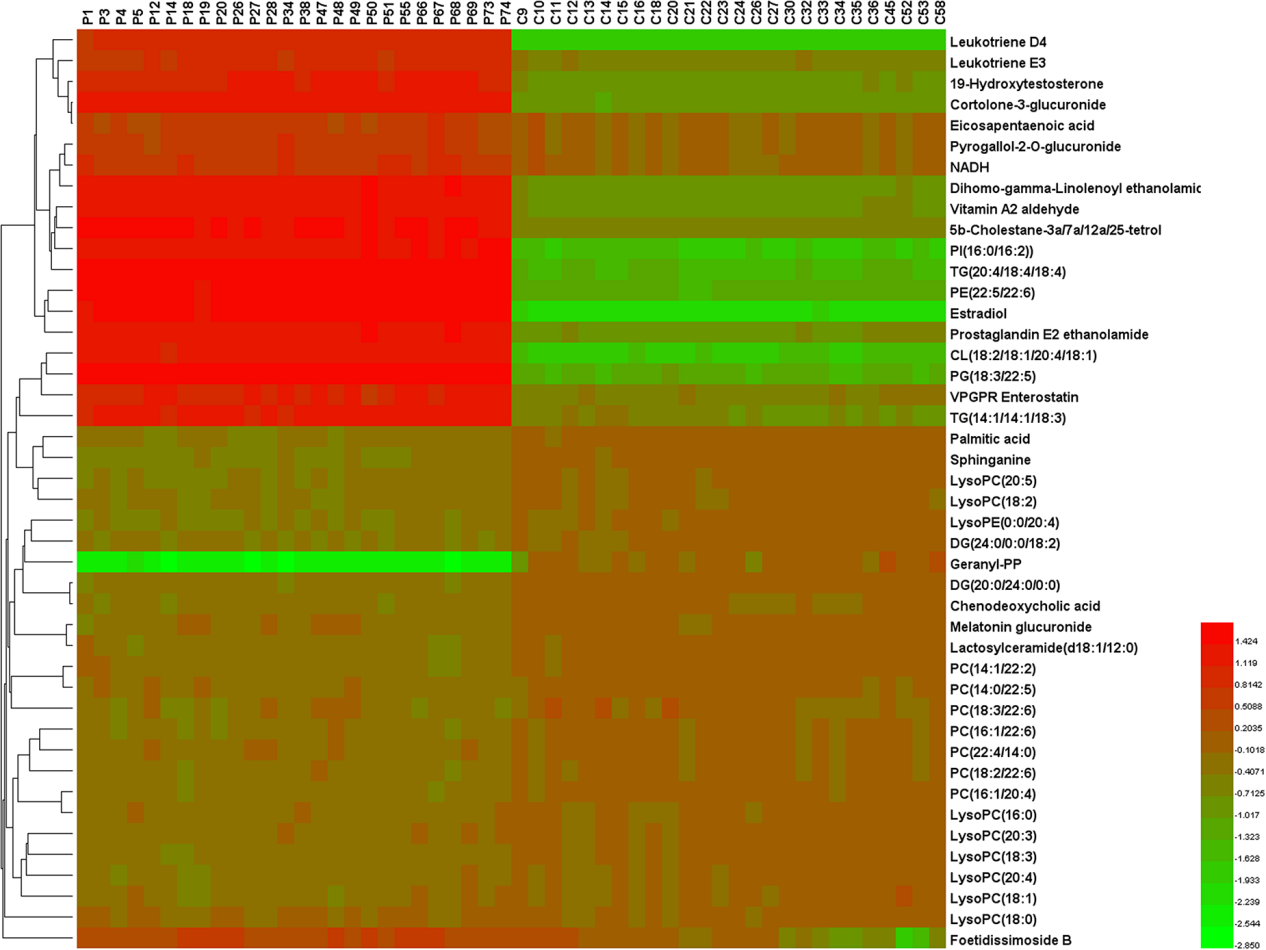
Heat map of the 44 differential metabolites (Psoriasis patients are labelled P... and healthy controls are labelled C...). The ratio of metabolite in the subject samples to the average of those in the healthy control samples was calculated, and then the metabolic alteration was demonstrated as log10 (ratio), the major metabolic alterations in psoriasis were visualized in a heat map plot.

In this study 5220 compounds obtained peak detection were processed by PLS-DA, 44 significant difference metabolites in comparison of psoriasis patients and healthy controls were screened out to be the potential biomarkers, the fold changes and P-value between the PV and HC samples were calculated and shown in Table 1, involved in glycerophospholipid metabolism, sphingolipid metabolism, arachidonic acid metabolism, bile acid biosynthesis, these might be associated with the pathogenesis of psoriasis. The peaks intensities of potential biomarkers in serum of the two groups were shown in Figure 4. The ratio of metabolite in the subject samples to the average of those in the healthy control samples was calculated, and then the metabolic alteration was demonstrated as log10 (ratio), the major metabolic alterations in psoriasis were visualized in a heat map plot (Figure 5). The upregulation and downregulation of metabolites were illustrated clearly in Figure 5.

**Table 1:** Potential biomarkers and their metabolic pathways

No.	m/z	Identify Result	Element composing	Ion	HMDB ID	KEGG ID	Class	Fold Change	p-value	Trend ^a^
1	497.293	Leukotriene D4	C25H40N2O6S	M+H	HMDB03080	C05951	Fatty Acyls	8.4	2.34E-46	↑
2	442.258	Leukotriene E3	C23H39NO5S	M+H	HMDB02355	-	Fatty Acyls	6.9	5.55E-57	↑
3	627.442	Eicosapentaenoic acid	C20H30O2	2M+Na	HMDB01999	C06428	Fatty Acyls	3.7	4.95E-43	↑
4	394.270	Dihomo-gamma-Linolenoylethanolamide	C22H39NO2	M+2Na-H	HMDB13625	C13828	Fatty Acyls	20.9	1.94E-80	↑
5	274.274	Palmitic acid	C16H32O2	M+NH4	HMDB00220	C00249	Fatty Acyls	0.5	4.48E-39	↓
6	396.273	Prostaglandin E2 ethanolamide	C22H37NO5	M+H	HMDB13038	-	Fatty Acyls	21.9	6.82E-81	↑
7	806.565	Lactosylceramide (d18:1/12:0)	C42H79NO13	M+H	HMDB04866	C01290	Sphingolipids	0.5	9.96E-40	↓
8	302.305	Sphinganine	C18H39NO2	M+H	HMDB00269	C00836	Sphingolipids	0.4	6.84E-56	↓
9	542.322	LysoPC (20:5)	C28H48NO7P	M+H	HMDB10397	C04230	Glycerophospholipids	0.4	2.15E-47	↓
10	496.340	LysoPC (16:0)	C24H50NO7P	M+H	HMDB10382	C04230	Glycerophospholipids	0.6	1.28E-28	↓
11	524.373	LysoPC (18:0)	C26H54NO7P	M+H	HMDB10384	C04230	Glycerophospholipids	0.9	6.83E-06	↓
12	522.358	LysoPC (18:1)	C26H52NO7P	M+H	HMDB02815	C04230	Glycerophospholipids	0.7	8.31E-18	↓
13	520.340	LysoPC (18:2)	C26H50NO7P	M+H	HMDB10386	C04230	Glycerophospholipids	0.5	4.50E-36	↓
14	518.323	LysoPC (18:3)	C26H48NO7P	M+H	HMDB10387	C04230	Glycerophospholipids	0.5	1.75E-44	↓
15	546.352	LysoPC (20:3)	C28H52NO7P	M+H	HMDB10393	C04230	Glycerophospholipids	0.7	6.51E-28	↓
16	544.339	LysoPC (20:4)	C28H50NO7P	M+H	HMDB10395	C04230	Glycerophospholipids	0.5	3.41E-27	↓
17	502.293	LysoPE (0:0/20:4)	C25H44NO7P	M+H	HMDB11487	-	Glycerophospholipids	0.4	1.03E-37	↓
18	828.556	PC (18:3/22:6)	C48H78NO8P	M+H	HMDB08189	C00157	Glycerophospholipids	0.6	1.06E-10	↓
19	780.552	PC (14:0/22:5)	C44H78NO8P	M+H	HMDB07890	C00157	Glycerophospholipids	0.7	6.94E-15	↓
20	784.584	PC (14:1/22:2)	C44H82NO8P	M+H	HMDB07921	C00157	Glycerophospholipids	0.6	8.17E-32	↓
21	804.552	PC (16:1/22:6)	C46H78NO8P	M+H	HMDB08023	C00157	Glycerophospholipids	0.5	1.15E-32	↓
22	830.567	PC (18:2/22:6)	C48H80NO8P	M+H	HMDB08156	C00157	Glycerophospholipids	0.6	4.24E-25	↓
23	782.566	PC (22:4/14:0)	C44H80NO8P	M+H	HMDB08623	C00157	Glycerophospholipids	0.6	1.50E-20	↓
24	766.575	PC (16:1/20:4)	C44H80NO7P	M+H	HMDB13415	-	Glycerophospholipids	0.5	3.77E-35	↓
25	819.506	PG (18:3/22:5)	C46H75O10P	M+H	HMDB10673	-	Glycerophospholipids	40.2	1.02E-81	↑
26	807.496	PI (16:0/16:2)	C41H75O13P	M+H	HMDB09780	C00626	Glycerophospholipids	23.3	5.62E-84	↑
27	838.542	PE (22:5/22:6)	C49H76NO8P	M+H	HMDB09639	C00350	Glycerophospholipids	38.5	5.87E-80	↑
28	746.525	CL (18:2/18:1/20:4/18:1)	C84H148O17P2	M+2H	HMDB58795	-	Glycerophospholipids	16.7	6.58E-67	↑
29	769.605	TG (14:1/14:1/18:3)	C49H84O6	M+H	HMDB47894	-	Triacylglycerols	15.5	4.00E-69	↑
30	971.594	TG (20:4/18:4/18:4)	C59H90O6	M+2K+H	HMDB54281	-	Triacylglycerols	41.9	5.62E-89	↑
31	371.317	DG (24:0/0:0/18:2)	C46H86O5	M+H+Na	HMDB56120	-	Glycerolipids	0.4	3.87E-39	↓
32	369.352	DG (20:0/24:0/0:0)	C47H92O5	M+2H	HMDB07383	-	Glycerolipids	0.5	5.87E-42	↓
33	305.184	19-Hydroxytestosterone	C19H28O3	M+H	HMDB06769	C05294	Steroids and steroid derivatives	12.3	1.61E-63	↑
34	475.310	5b-Cholestane-3a,7a,12a,25-tetrol	C27H48O4	M+K	HMDB00524	-	Steroids and steroid derivatives	27.3	5.70E-92	↑
35	543.293	Cortolone-3-glucuronide	C27H42O11	M+H	HMDB10320	-	Steroids and steroid derivatives	18.1	1.91E-76	↑
36	586.381	Estradiol	C18H24O2	2M+ACN+H	HMDB00151	C00951	Steroids and steroid derivatives	36.9	3.86E-73	↑
37	666.137	NADH	C21H29N7O14P2	M+H	HMDB01487	C00004	(5’->5’)-dinucleotides	5.4	3.46E-43	↑
38	393.298	Chenodeoxycholic acid	C24H40O4	M+H	HMDB00518	C02528	Bile acids, alcohols and derivatives	0.5	1.39E-33	↓
39	627.109	Pyrogallol-2-O-glucuronide	C12H14O9	2M+Na	HMDB60017	-	Carbohydrates and carbohydrate conjugates	4.5	1.34E-41	↑
40	685.842	Foetidissimoside B	C63H100O31	M+H+NH4	HMDB36252	-	Prenol Lipids	2.8	7.43E-21	↑
41	315.080	Geranyl-PP	C10H20O7P2	M+H	HMDB01285	C00341	Prenol Lipids	0.0	2.38E-32	↓
42	283.201	Vitamin A2 aldehyde	C20H26O	M+H	HMDB35695	C05918	Prenol lipids	19.9	4.61E-78	↑
43	409.161	Melatonin glucuronide	C19H24N2O8	M+H	HMDB60830	-	Nucleoside and nucleotide analogues	0.6	2.62E-24	↓
44	525.304	VPGPR Enterostatin	C23H40N8O6	M+H	HMDB03577	-	Carboxylic acids and derivatives	12.4	4.26E-69	↑

44 significant difference metabolites were screened out to be the potential biomarkers according to the value of variable importance for projection (VIP) value in PLS-DA model, the fold changes and P-value between the PV and HC samples were calculated. These potential biomarkers were involved in glycerophospholipid metabolism, sphingolipid metabolism, arachidonic acid metabolism, bile acid biosynthesis.

a Trend change compared with healthy controls (HC). (↑) and (↓) represent the corresponding compound is up- regulated and down-regulated in PV patients compared with the HC, respectively.

LysoPC: Lysophosphatidylcholine

LysoPE: Lysophosphatidyl ethanolamine

PC: Phosphatidylcholine

PG: Phosphatidylglycerol

PI: Phosphatidylinositol

PE: Phosphatidylethanolamine

CL: Cardiolipin

TG: Tracylglycerol

DG: Diacylglycerol

NADH: Nicotinamide adenine dinucleotide

VPGPR: Val-Pro-Gly-Pro-Arg

### Metabolic pathway analysis

**Figure 6:**
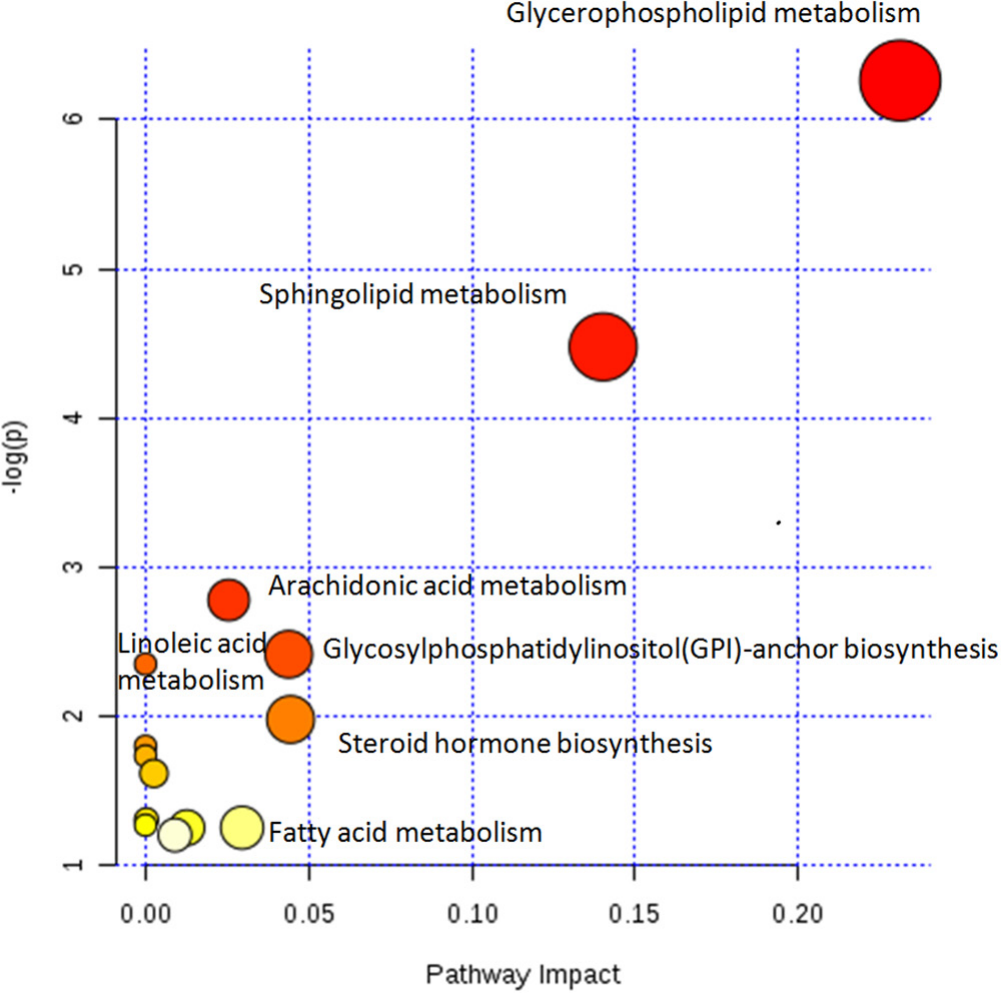
Disturbed metabolic pathways showed various metabolism changes when comparing psoriasis and control The metabolic pathway analysis was an integrating enrichment analysis based on the human metabolic pathways. The different color and size of the symbol means the different level of significance, there were more potential biomarkers in the data were involved in the pathway, the color was darker or the size was larger. Glycerophospholipid metabolism, sphingolipid metabolism, arachidonic acid metabolism was most relevant pathways in psoriasis metabolism.

The metabolic pathway analysis was an integrating enrichment analysis based on the human metabolic pathways. The visualized result facilitated further biological interpretation to reveal the most relevant pathways as shown in Figure 6. The different color and size of the symbol means the different level of significance, there were more potential biomarkers in the data were involved in the pathway, the color was darker or the size was larger. As Figure 6 shown, the glycerophospholipid metabolism, sphingolipid metabolism, arachidonic acid metabolism was most relevant pathways in psoriasis metabolism.

### Correlation network

**Figure 7:**
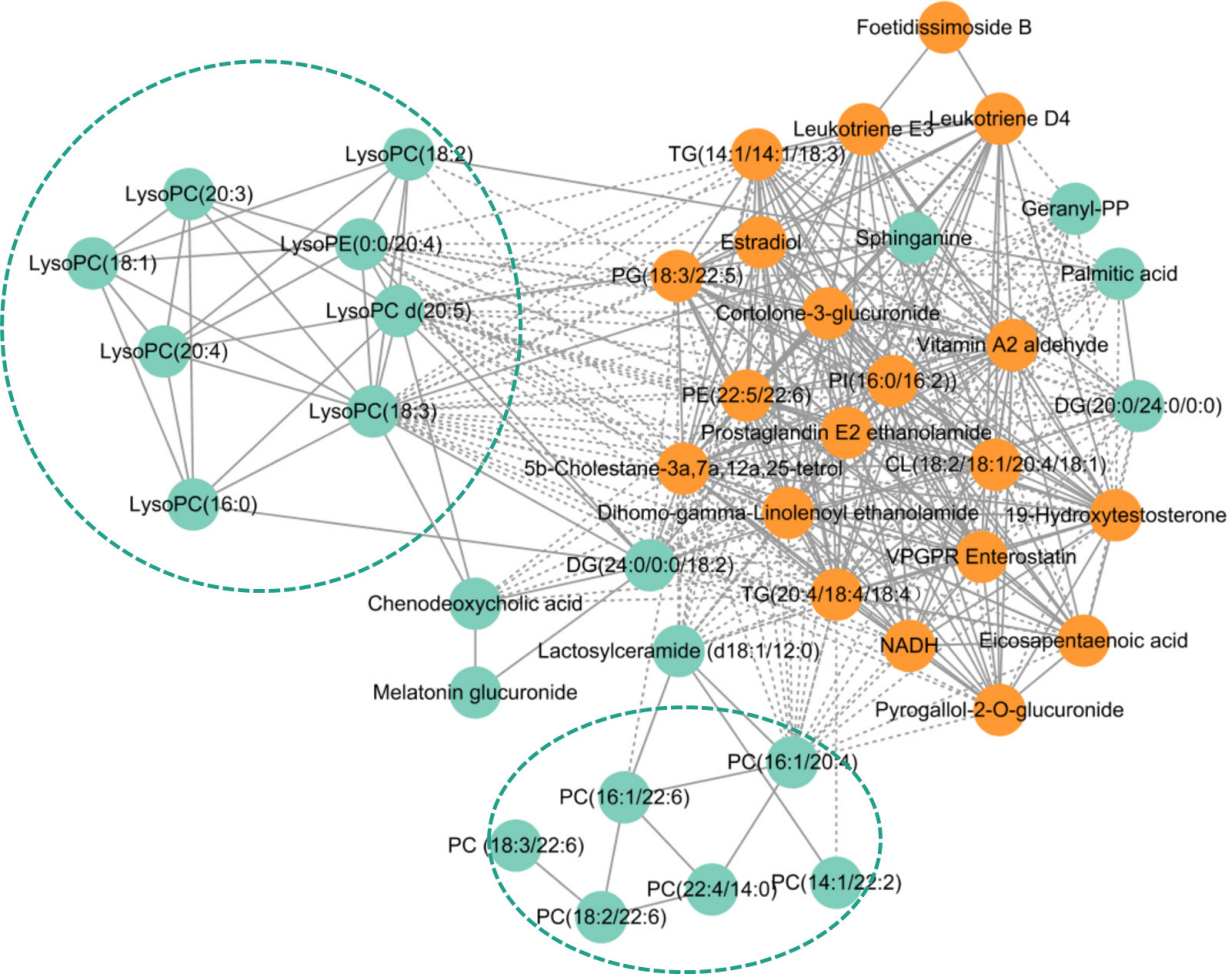
Pearson correlation network of metabolites Pearson correlation coefficients between the 44 metabolites were calculated on the basis of the average normalized quantities of metabolites. Highly correlated metabolites with r> 0.8 were connected with solid lines, which metabolite with r< -0.8 were connected with dotted lines in the network. Orange plots indicated up-regulated metabolites and green plots indicated down-regulated metabolites in PV.

To investigate the latent relationships of the 44 metabolites, the Pearson correlation coefficients between the metabolites were calculated on the basis of the average normalized quantities of metabolites. Highly correlated metabolites with r> 0.8 were connected with solid lines, which metabolite with r< -0.8 were connected with dotted lines in the network (Figure 7). Orange plots indicated up-regulated metabolites and green plots indicated down-regulated metabolites in PV.

Most metabolites in lysophospholipid and phospholipid were down-regulated in PV groups and had highly correlated coefficients. Levels of some correlated arachidonic acid, inflammatory factor, sphingolipid were up-regulated.

### Receiver operating characteristic curve

**Figure 8:**
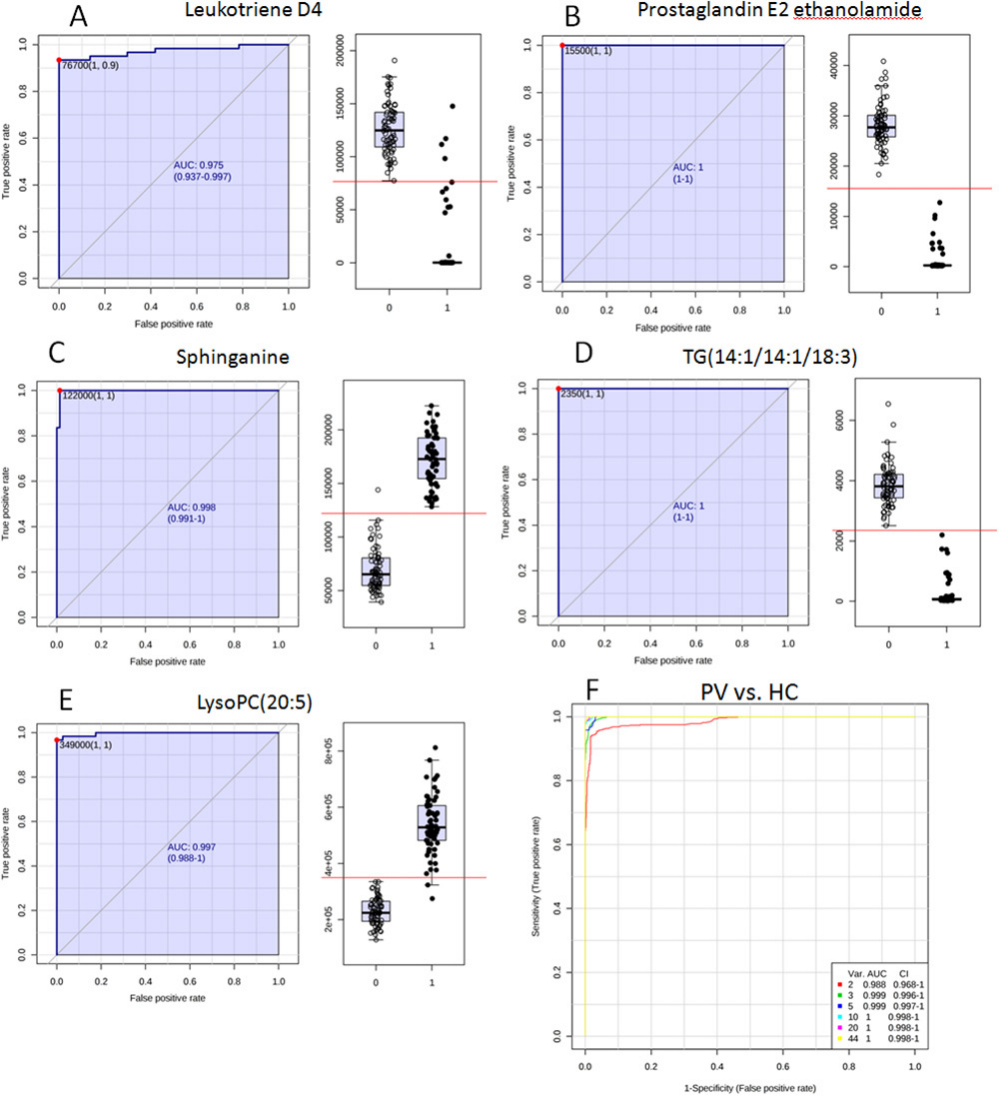
The typical ROC curve plots of potential biomarkers **(A~E)** Typical ROC curves and AUC values of potential biomarkers with high-performance prediction; **(F)** Multivariate exploratory ROC analysis overview.

ROC curve analysis is generally considered to be the gold standard for the assessment of biomarker performance. The results of ROC curve analysis of the 44 differentiated metabolites guaranteed the reliability of potential biomarkers for wide and qualified independent validation. The value of area under the curve (AUC) was higher than 0.7 suggested good predict ability of potential biomarkers. Typical ROC curves and AUC values were shown in Figure 8A~8E. Multivariate exploratory ROC analysis overview was shown in Figure 8F.

## DISCUSSION

We developed an untargeted metabonomics method to investigate the pathogenesis of psoriasis, 44 potential biomarkers were screened out, there involved in several metabolic pathways.

### Arachidonic acid metabolism

Leukotrienes are a family of eicosanoid inflammatory mediators produced in leukocytes by the oxidation of arachidonic acid by the enzyme arachidonate 5-lipoxygenase. Leukotrienes can regulate immune responses by using lipid signaling to convey information to either the cell producing them (autocrine signaling) or neighboring cells (paracrine signaling). The product of Leukotriene such as histamine and prostaglandins are also the inflammatory mediators.

Leukotriene D4 (LTD4) is a potent inflammatory mediator. It is also a pro-inflammatory mediator present at high levels in many inflammatory diseases, caused increasing risk for subsequent cancer development in chronic inflammation. LTD4 have been mentioned to associate with the pathogenesis of several inflammatory disorders, such as asthma and inflammatory bowel disease.

Leukotriene E3 is an eicosanoid derived from 8,11,14-eicosatrienoic acid by the 5-lipoxygenase-leukotriene pathway. Leukotriene E3 is also a by-product of the metabolism of leukotriene C3. The eicosanoids are a diverse family of molecules consists of the prostaglandins (PGs), thromboxanes (TXs), leukotrienes (LTs) and lipoxins (LXs) have powerful effects on cell function. The eicosanoids had been known that they were involved in asthma, pain, fever and vascular responses, present evidence indicates that eicosanoids exert relevant effects on immune, as well as structural, cells pertinent to fibrogenesis [39–42].

Our result revealed that the content of Leukotriene D4 and Leukotriene E3 in serum was significantly higher in psoriasis patients than that in healthy controls. Arachidonic acid metabolism abnormality was an important factor in pathogenesis of psoriasis. This result is consistent with our previous studies on urine metabolome of psoriasis patients [37].

### Lipid metabolism

In this study, some lipid compounds were significant different between PV and HC, involved in glycerophospholipid metabolism, Phospholipid Biosynthesis and sphingolipid metabolism.

In recent years, more and more lipid metabolism disorders in psoriasis have been reported. There was a significant relationship between severity of psoriasis and serum lipid profile. This may elevate the risk of atherosclerosis, particularly cardiovascular disorders [43, 44]. Epidemiological studies and clinical investigation research showed patients had high prevalence of cardiovascular comorbidities, and high cardiovascular risk according to the Framingham risk score [45–47].

Sphingolipids have important biological and structural functions in the maintenance of the skin barrier function and regulate cellular processes including proliferation, differentiation and apoptosis of keratinocytes. Previous studies results showed that the composition change and metabolism disorder of epidermal sphingolipids were happened in many dermatologic diseases such as psoriasis, atopic dermatitis and ichthyoses [48].

The prostanoids and leukotrienes as the lipid mediators are metabolites of arachidonic acid mediated through their respective receptors expressed on target cells and released in various pathophysiological conditions. It has been proved that the balance of the production and the receptor expression of each lipid mediator are important for the homeostasis maintaining in our body. The functions of prostanoids and leukotrienes in skin inflammatory diseases focusing on contact dermatitis, atopic dermatitis, and psoriasis had been reviewed [49–51].

Therefore, the lipid compounds may to be the diagnostic and examination index in psoriasis in future.

### Bile acid biosynthesis

Chenodeoxycholic acid is a bile acid which can facilitate excretion, absorption, and transport of fats and sterols in the intestine and liver, usually considered to be a physiological detergent. Bile acids are also the steroidal amphipathic molecules derived from the catabolism of cholesterol. They are essential for the absorption of dietary fats and vitamins, participate in the regulation of all the key enzymes involved in cholesterol homeostasis, and also modulate bile flow and lipid secretion. The unique detergent properties of bile acids are essential for the digestion and intestinal absorption of hydrophobic nutrients [52–55]. In this study, the content of chenodeoxycholic acid in PV was lower than HC. The deficiency of bile acids might involve in the pathogenesis of psoriasis [56, 57].

In this study, an untargeted high-throughput liquid chromatography coupled to mass spectrometry method was carried out for the global metabonomics analysis with the purpose of finding out more potential biomarkers in human serum for diagnosing psoriasis vulgaris patients and exploring pathogenesis of psoriasis. The levels of the 44 potential biomarkers demonstrated there were significant differences between PV patients and HC, the psoriasis patients groups with different severity also showed the different metabolic trajectory. These 44 potential biomarkers provided a better comprehension of the pathophysiological progress of PV, indicated the psoriasis may involve in several disturbed metabolic pathways including glycerophospholipid metabolism, sphingolipid metabolism, arachidonic acid metabolism, bile acid biosynthesis. Furthermore, this study was an exploratory research, these potential biomarkers still need repeated verified by more clinical samples and quantitative analysis by standards. To illustrate the pathogenesis of psoriasis systematically and comprehensively, the metabonomics results should be integrated and compared with the outcomes of other ‘omics’ studies such as transcriptomics, proteomics and genomics, it will to be benefit to discover the biomarkers group, and apply them to real clinical disease diagnostics and treatments.

## MATERIALS AND METHODS

### Human serum collection

75 out patients confirmed psoriasis vulgaris were recruited from the dermatology department of Guangdong provincial hospital of Chinese medicine (Guangzhou, Guangdong, China) and dermatology department of people’s liberation army 195 hospital (Xianning, Hubei, China). 75 healthy volunteers were recruited from physical examination department of Guangdong provincial hospital of Chinese medicine. Clinical characteristics of patient and controls were described as Table 2. The study protocol (No. B2010-08-01) was approved by the institutional ethics committee of Guangdong provincial hospital of Chinese medicine, and all of the patients in this study have understood and signed informed consent.

**Table 2:** Clinical characteristics of psoriasis patients and healthy controls

Characteristics	PV Patients	Healthy Controls
Number of subjects	75	75
Gender (male/female)	50/25	47/28
Age	36.7±13.1	33.3±12.2
PASI score		
mild (PASI score≤10)	37	-
moderate to severe (PASI score>10)	38	-

The clinical characteristics of 75 psoriasis vulgaris patients and 75 healthy volunteers were described.

All individuals were asked to be on empty stomach for 8-14 hours, and refrained from drinking, smoking for 24 hours before serum collection. Serum was prepared by evacuated and promoting coagulating tubes and centrifugation (3000 rpm, 10min). The upper serum layer was stored at -80°C until sample analysis.

### Reagents and instrumentations

Acetonitrile (HPLC grade) was purchased from Merck (Darmstadt, Germany). Formic acid (analytical grade) was purchased from Fluka (Buchs, Switzerland). Ultrapure water (18.2 MΩ) was prepared by using a Milli-Q water purification system (Millipore, Bedford, MA, USA).

Serum metabolic profiling data were acquired by an ultra-performance liquid chromatography (Waters ACQUITY UPLC, USA) coupled with a quadruple-time-of-flight mass spectrometry (AB SCIEX Triple-TOF 5600, USA). Vortex mixer was purchased from Qilinbeier (VORTEX-5, Jiangsu, China), Centrifuge was purchased from Beckman Coulter (AllegraTM X-22, Beckman Coulter Corporation, USA).

### Sample preparation

The frozen serum samples thawed at 4°C, 100μL serum was mixed with 400μL acetonitrile and vortexed for 2min, then centrifuged at 13,000rpm for 20 min at 4°C. The supernatant was collected and transferred to a 96 well collection plate waiting for LC-MS analysis.

### LC-MS analysis

Chromatography was carried out by an ACQUITY BEH C18 chromatography column (100× 2.1 mm, 1.7 μm, Waters). The mobile phases consisted of ultra-pure water with 0.1% formic acid (phase A) and acetonitrile with 0.1% formic acid (phase B). A linear gradient elution was carried out. At first, 2% mobile phase B was run for 1min, then quickly increased to 15% in 1min and kept for 5min, followed by a linear increase to 65% mobile phase B from 7 to 15 min, and then further increased to 95% mobile phase B from 15 to 23 min, kept 95% for 8min, at last the proportion of phase B returned to 2% in 0.1min, and the column was allowed to re-equilibrate for 2min before the next injection. The column temperature was maintained at 40°C. The flow rate was 0.4mL/min. The temperature of auto-sampler was maintained at a 4°C for the duration of analysis, and the sample injection volume was 2μL.

The data were acquired from m/z 100 to 1000 in positive electrospray ionization (ESI+) mode. The ion source voltage flow (ISVF) was set to 5500, source temperature was set to 650°C. The information dependent acquisition (IDA) function was enabled to obtain the high resolution, high mass accuracy MS/MS spectra. All the acquired m/z data were real-time corrected using an independent reference to ensure the higher accuracy.

### Raw data processing and statistical analysis

The raw spectrometric data were collected by Analyst^®^ TF Software Kit (Version 1.5.1, AB SCIEX, USA), and processed by the MarkerView™ software (Version 1.2.1, AB SCIEX, USA) for chromatography peak recognition and matching, noise filtering, data normalization, peak list data were loaded to Simca-P software (version 14.1, Umetrics, Umeå, Sweden) for multivariate data analysis. Principal components analysis (PCA), partial least squares discriminate analysis (PLS-DA) and orthogonal signal correction partial least squares discriminate analysis (OPLS-DA) was applied to distinguish psoriasis vulgaris (PV) patients from healthy controls (HC). The metabolites with the variable importance in the projection (VIP) values > 1 in the PLS-DA model were selected as candidate potential biomarkers. The P-value cut-off in t-test was set to 0.05 [58]. Furthermore, the generalization ability of fitting models was evaluated by 200 permutations of cross-test validation. As the LC-MS analysis provided the retention time, precise molecular mass and MS/MS information of the metabolites, potential biomarkers could be identified. We screened out the candidate by PeakView® Software (Version 1.2, AB SCIEX, USA), current free metabolite databases such as The Human Metabolome Database (HMDB, http://www.hmdb.ca/), KEGG (http://www.genome.jp/kegg) were used to search for molecule formula and structures [59–61]. A heat map was plotted to illustrate up-regulations and down-regulations in significant change metabolites by using Heml 1.0 (Heat map illustrator). A correlation network was plotted by Cytoscape software package version 3.2.1 (National Institute of General Medical Sciences, Bethesda, MD, USA). MetaboAnalyst 3.0 (www.metaboanalyst.ca) was applied to draw the receiver operating characteristic (ROC) curves of the potential biomarkers [62]. MetaboAnalyst 3.0 was also applied to analyze pathways for discovering the most important metabolic pathway which significant metabolites enriched in.

Thanks for the cooperation of People’s Liberation Army 195 Hospital, Xianning, Hubei, China.
